# Institutional Readiness and Diagnostic Challenges for the Management of Pyrexia of Unknown Origin (PUO) in Nepal: A Mixed-Methods Study at Tertiary Level Hospitals

**DOI:** 10.21203/rs.3.rs-8662503/v1

**Published:** 2026-02-22

**Authors:** Birendra Gupta, Chandramani Wagle, Nikita Acharya, Abhay Kumar Sah, Jyoti Takanche, Rajeev Shrestha, Bimal Sharma Chalise, Pradip Gyanwali, Madhusudan Subedi, Tracy Hazen, Man Charurat

**Affiliations:** 1 Institute of Human Virology, University of Maryland School of Medicine, Baltimore, MD USA; 2 Global Clinical Research, Kathmandu, Nepal; 3 Center for Infectious Disease Research and Surveillance, Dhulikhel Hospital, Kathmandu University Hospital, Dhulikhel, Nepal; 4 Sukraraj Tropical and Infectious Disease Hospital, Kathmandu, Nepal; 5 Madan Bhandari Academy of Health Science, Hetauda, Nepal; 6 School of Public Health, Patan Academy of Health Sciences, Patan, Nepal; 7 Center for Advanced Microbiome Research and Innovation, Institute for Genome Sciences, University of Maryland School of Medicine, Baltimore, MD, USA; 8 Department of Microbiology and Immunology, University of Maryland School of Medicine, Baltimore, MD, USA; 9 Department of Medicine, University of Maryland School of Medicine, Baltimore, MD USA; 10 Department of Epidemiology and Public Health, University of Maryland School of Medicine, Baltimore, MD USA

**Keywords:** Pyrexia of Unknown Origin, Institutional Readiness, Diagnostics Gap, Mixed-Methods, Health Systems Strengthening, Nepal

## Abstract

**Background:**

Pyrexia of Unknown Origin (PUO) remains a significant diagnostic challenge in low-resource settings, where limited laboratory capacity and fragmented health systems impede timely etiologic identification. In Nepal, evidence on institutional readiness, diagnostic capacity, and system-level barriers for PUO care is limited, hindering standardized clinical decision-making and timely diagnosis. This study assessed the diagnostic capacity, workforce expertise, and governance structures for PUO management across Nepal.

**Methods:**

A concurrent mixed-methods study was conducted across 11 tertiary care hospitals in six provinces of Nepal. Quantitative data on governance, service delivery, diagnostics, human resources, infrastructure, and financing were collected using standardized facility assessment tools and descriptively analyzed using SPSS version 23. Qualitative data were obtained from 33 key informant semi-structured interviews (hospital administrators, clinicians, and laboratory personnel), transcribed verbatim, and subjected to thematic analysis in NVivo with intercoder reliability assessment. Findings were integrated through triangulation.

**Results:**

Quantitative assessments revealed limited institutional preparedness for PUO management, characterized by the absence of dedicated clinical guidelines, formal referral pathways, and designated focal points in most hospitals. Basic diagnostic services were widely available; however, access to advanced molecular diagnostics was inconsistent and often dependent on external laboratories, contributing to diagnostic delays. Qualitative findings contextualized these gaps, highlighting fragmented governance, weak interdisciplinary coordination, and heavy reliance on empirical treatment of pyrexia, frequently influenced by prior antibiotic exposure. Human resource constraints were prominent, particularly shortages of infectious disease specialists, pediatric expertise, microbiologists, and trained laboratory personnel. Infrastructure limitations, inefficient referral mechanisms, and substantial out-of-pocket expenditures further constrained optimal care. Digital health tools, including electronic medical records and telemedicine, were inconsistently implemented despite being viewed as potential facilitators of improved coordination and follow-up.

**Conclusions:**

Management of PUO in Nepal is limited by system-level weaknesses in governance, diagnostic capacity, workforce skills, financing, and digital health integration. Closing these gaps through standardized clinical guidelines, strengthened laboratory systems, focused workforce training, improved referral pathways, and strategic digital health investments could shorten diagnostic timelines, improve patient outcomes, and advance national priorities such as antimicrobial stewardship and epidemic preparedness.

## Background

Pyrexia of Unknown Origin (PUO) remains a formidable diagnostic challenge in clinical medicine, characterized by prolonged fever that evades diagnosis despite initial investigation [1]. First defined by Petersdorf and Beeson in 1961, PUO is characterized by a temperature exceeding 38.3°C (101°F) on multiple occasions, lasting for more than three weeks, with no diagnosis reached after one week of intensive hospital evaluation [2]. The etiological spectrum is broad, encompassing infectious, inflammatory, neoplastic, and miscellaneous disorders, with the relative prominence of each category varying significantly between high-income countries and low- and middle-income countries (LMICs) [3–5]. Despite advances in diagnostic imaging and microbiological techniques, the relative prominence of each category has changed over time, with an increasing proportion of patients remaining undiagnosed, which may be as high as 51% of cases [6]. Infectious causes account for 17–35% of cases, inflammatory causes 24–36%, neoplastic causes 10–20%, and miscellaneous causes 3–15% [6–8]. In resource-constrained countries, the infectious etiology of PUO is most prevalent; in developed countries, PUO is likely due to non-infectious inflammatory disease [6, 9]. A recent (2025) meta-analysis of Acute Febrile Illness (AFI) etiologies in LMICs reported that 36.9% of cases are classified as fever of unknown origin, reflecting critical gaps in pathogen detection [10]. Studies have documented PUO outbreaks across multiple regions, particularly during seasonal transitions, with reported morbidity and mortality affecting both pediatric and adult populations [4, 11]. Previous investigations have reported that more than 60% of febrile illnesses in resource-limited settings remain undiagnosed, reflecting constrained diagnostic capacity and fragmented testing pathways, substantially higher than global estimates, where approximately 25–50% of PUO cases lack a definitive diagnosis [12–14].

Despite the established clinical burden of PUO, the broader institutional and systemic factors that contribute to diagnostic failure remain largely unexplored in resource-limited settings [15]. While the transition from conventional hematologic and serologic assays to molecular diagnostics like PCR and metagenomic Next-Generation Sequencing (mNGS) has improved diagnostic yields globally, these advancements have not been uniformly integrated into the healthcare infrastructure of LMICs like Nepal [4, 16–19]. However, even in high-income settings, such technologies are rarely available as routine in-house diagnostics and are commonly accessed through referral to specialized or accredited external laboratories due to high costs, infrastructure requirements, and the need for specialized expertise [20]. Over-reliance on empirical syndromic treatment promotes antimicrobial misuse, fueling resistance, while surveillance gaps hinder recognition of novel or re-emerging pathogens [21]. It is currently unknown how institutional preparedness encompassing laboratory capacity, workforce availability, and standardized referral pathways directly influences the diagnostic trajectory of PUO patients in LMICs including Nepal. Most existing literature emphasizes patient-level pathogen detection rather than healthcare facility readiness, leaving unclear why many patients are discharged without a definitive diagnosis and underscoring systemic gaps in diagnostic processes.

Studies have critically analyzed the institutional shortcomings for managing orthopedic infections, such as inadequate diagnostic equipment or the lack of specialized human resources that drive this reliance on empirical therapy [22]. Moreover, while stakeholder perspectives are crucial for understanding implementation barriers, mixed-methods research integrating facility assessments with qualitative insights on diagnostic challenges is absent from the Nepalese context [23, 24]. Without addressing these systemic gaps, improvements in bedside care remain disconnected from the realities of the healthcare infrastructure.

This mixed-methods study addresses these gaps by assessing institutional readiness and diagnostic challenges for PUO management across tertiary care hospitals in multiple provinces of Nepal. We conducted facility evaluations at tertiary-level hospitals to identify gaps in equipment, laboratory services, human resources, and treatment protocols, while exploring stakeholder perspectives on implementing advanced diagnostics. The rationale for this study is to generate actionable evidence for policymakers and health administrators to guide targeted interventions, resource allocation, and capacity-building initiatives. Ultimately, this research seeks to inform strategies that enhance diagnostic accuracy, guide antimicrobial use, and improve the management of persistent and undiagnosed febrile illness in Nepal.

## Methods

### Study Design and Setting

**Figure 1.**
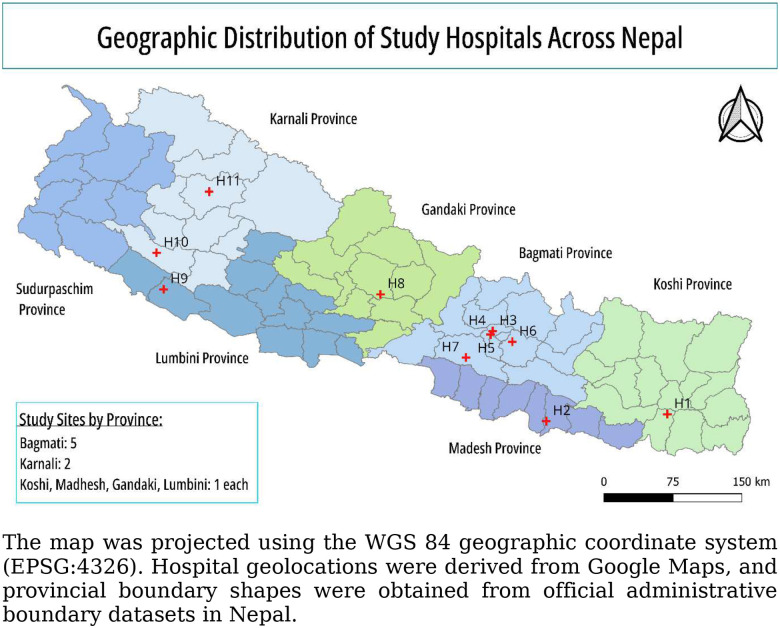
Geographic Distribution of Selected Study Sites (Hospitals)

A concurrent, cross-sectional, mixed-methods study was conducted to assess institutional readiness and diagnostic challenges for the management of PUO. Quantitative facility assessments and qualitative semi-structured key informant interviews (KIIs) were conducted in parallel and integrated during analysis to provide a comprehensive understanding of diagnostic capacity, institutional practices, and system-level barriers. Nepal has a large public health network comprising more than 8,000 public hospitals; however, only a small proportion function as tertiary or specialized referral centers. As of fiscal year 2081/82, fewer than 100 hospitals nationwide are classified as general hospitals (100–300 beds), specialized hospitals (≥100 beds), or super-specialty hospitals (≥50 beds), which collectively serve as referral sites for complex conditions such as PUO. Bagmati Province, the country’s primary referral hub, disproportionately concentrates these higher-level hospitals. Therefore, we included a larger number of study sites from Bagmati Province, reflecting its central role in advanced diagnostic and referral care [25]. This study was conducted in 11 tertiary-level hospitals across six provinces of Nepal (Koshi, Madhesh, Bagmati, Gandaki, Lumbini, and Karnali) and included academic, government, and non-profit referral institutions (Figure 1). Hospitals were selected to ensure broad geographic representation and diversity in service delivery, infrastructure, patient volume, and diagnostic capacity. Data collection occurred between September 18, 2025, and November 27, 2025.

### Study Population and Sampling

The units of analysis were the healthcare institutions and the key professionals involved in PUO management within them. At each hospital, data were collected through a facility audit and semi-structured key informant interviews (KIIs). Participants for KIIs were purposively selected based on their direct involvement in the diagnosis, management, or administrative oversight of PUO cases and had at least one year of experience in their current role. This included clinicians (e.g., internists, pediatricians, and infectious disease specialists), laboratory personnel (e.g., microbiologists and lab supervisors), and senior hospital administrators.

A purposive sampling strategy was used to select hospitals based on the following criteria: (a) recognition as a tertiary care or major referral center; (b) availability of basic laboratory and imaging diagnostics; and (c) institutional willingness to participate. Given the limited number of specialized and super-specialty hospitals nationally, purposive sampling was considered appropriate to capture institutional readiness at the highest level of care. Within each hospital, authorities were consulted to identify key informants and capture comprehensive perspectives. Potential selection bias may arise due to purposive selection of tertiary hospitals and availability-based participation of key informants.

### Study Variables

The study evaluated four primary domains of institutional readiness for PUO management, as summarized in Table 1. These domains were designed to capture both the structural and operational capacity of hospitals, as well as the perceived feasibility of adopting advanced diagnostics and innovations.

Basic diagnostics were widely available across hospitals and included routine laboratory and imaging tests that form the standard first-line evaluation for PUO, such as hematology, biochemistry, microscopy, culture, serology, and conventional imaging. Advanced diagnostics referred to more specialized laboratory assays, including PCR-based molecular tests and metagenomic next-generation sequencing, which are typically used for complex or undiagnosed cases and are often accessed through external referral mechanisms. Digital diagnostics encompassed hospital-wide electronic health records, laboratory information systems, and reporting platforms that support diagnostic decision-making, case tracking, and surveillance (Table 1).

### Data Collection Tools and Procedures

Data were collected using two primary data collection tools designed for quantitative and qualitative assessment. The first was a structured facility assessment form developed by the research team with references from established WHO frameworks, including the WHO Service Availability and Readiness Assessment (SARA) [26], WHO Laboratory Assessment Tool [27], and WHO Rapid Hospital Readiness Checklists [28]. The second instrument was a semi-structured interview guide, designed to obtain qualitative insights from key informants such as hospital administrators and clinical staff, and reviewed by subject-matter experts in infectious diseases and hospital administration.

Quantitative data were collected in person using the paper-based facility assessment forms administered by trained members of the research team, while qualitative data were collected through key informant interviews (KIIs) conducted either in person or online depending on participant availability. Interviews were audio-recorded with participant consent, lasted approximately 30–45 minutes, and were conducted in either English or Nepali according to participant preference. Both quantitative data and key informant questionnaire was developed for this study and is provided in supplementary file.

### Pre-testing, Validity, and Reliability

Data collection tools were pretested at a Kathmandu-based tertiary hospital not included in the study to refine clarity, cultural relevance, and feasibility. Content validity was established through expert review prior to ethical approval. Reliability of quantitative data was ensured through standardized training of data collectors and inter-rater verification of 10% of facility audit items. Qualitative rigor was enhanced through member checking, triangulation with quantitative findings, and assessment of intercoder consistency. Triangulation across data sources was used to reduce single-source bias.

### Data Management and Analysis

Quantitative data collected through paper-based facility assessment forms were reviewed for completeness at each study site, entered into Microsoft Excel, and cleaned prior to analysis. Descriptive analyses were performed using SPSS version 23, with results summarized as frequencies and percentages to characterize institutional readiness indicators and diagnostic practices. KII audio recordings were transcribed and translated into English to guarantee that participant replies were accurately represented. NVivo was used for coding and generating sub-themes and themes. Braun and Clarke’s six-step thematic analysis was applied for analyzing qualitative data [29]. To identify institutional obstacles, gaps, and possibilities in the management of PUO, initial codes were systematically generated and subsequently synthesized into sub-themes and themes as presented in Table 2. Intercoder reliability was assessed to enhance consistency and rigor in coding, ensuring that the interpretation of qualitative data was reproducible and credible. Supporting quotes were used in the analysis to add depth and preserve the “voice” of the participants. Potential response bias was mitigated by ensuring confidentiality, anonymization, and the use of neutral, non-evaluative questioning.

### Ethical Considerations

The study was conducted in accordance with the principles of the Declaration of Helsinki and received ethical approval from the Ethical Review Board of the Nepal Health Research Council [Ref. 269–2025] and the University of Maryland, Baltimore Institutional Review Board [Ref. HP-00116457].

Prior to ethical approval, institutional site acceptance letters were obtained from all participating hospitals and submitted to NHRC as part of the review process. Informed consent was obtained from all interview participants prior to data collection. Written consent was obtained for in-person interviews, while verbal consent was obtained for online interviews where written consent was not logistically feasible. This approach was approved by the ethics committee and ensured participant autonomy while minimizing administrative burden during remote data collection. All consent procedures were documented by the research team.

Participant identities and hospital names were de-identified using unique codes, and all audio recordings, transcripts, and datasets were securely stored with access restricted to the core research team. No patients, biological samples, or clinical interventions were involved in this study.

## Results

### Study Sites and Participants Characteristics

A total of 11 tertiary-level hospitals were included, primarily representing public academic institutions (6, 54.5%), followed by government public hospitals (3, 27.3%), and one each from the community and private academic sectors (2, 18.2%). Geographically, the study covered six provinces, with the highest concentration in Bagmati Province (5, 45%). No sites from Sudurpaschim Province were included during the study period due to difficulties in coordination with hospitals within the limited timeframe. A total of 33 key informant semi-structured interviews (KIIs) were conducted, with three per hospital (one hospital director/administrator, one infectious disease physician or clinician, and one laboratory technologist/microbiologist). Of the 33 participants, the majority (31, 94%) were male, and only 2 (6%) were female. Participants had substantial professional experience across clinical, laboratory, and administrative roles. Hospital and participant characteristics are summarized in Table 3.

### Health System Readiness for PUO Management

#### Governance, Policy, and Clinical Guidance

Quantitative findings indicate limited institutional governance mechanisms for PUO management across the 11 study sites (Table 4). PUO-specific clinical guidelines or standard operating procedures were available in only one hospital, and formal response drills or simulations had been conducted in a similarly small proportion. None of the hospitals reported having a standard operating procedure for internal coordination among departments for PUO case management. Designated PUO focal persons or committees were present in fewer than one-third of hospitals, suggesting minimal formalization of PUO governance structures.

Qualitative findings were consistent with these observations. Participants at every study site consistently reported that there was no nationally recognized protocol or guideline for the diagnosis and treatment of PUO.

There was no written, finalized Standard Operating Procedure (SOP) for PUO in any of the hospitals. One infectious disease consultant explicitly noted that *“there is no separate local or national guideline in Nepal for PUO cases,”* highlighting the complete lack of formal policy direction at both national and institutional levels.

Clinicians mostly used international textbooks and global algorithms, most frequently the Nelson Textbook of Pediatrics, to direct diagnostic routes in the absence of defined guidelines. Examinations were tailored to the patient’s history, place of origin, and suspected illness epidemiology. Clinical decision-making was primarily customized and clinician-driven. As one pediatric consultant explained, *“Rather than one rigid, well-defined protocol, the workup is individualized to the patient’s geography and specifics.”* Another consultant emphasized the lack of uniformity across clinicians, stating, *“We don’t have one single uniform checklist that everyone follows for every patient.”*

At the time of data collection, none of the PUO-related SOPs had been formally authorized or put into practice, although some hospitals stated that they were being discussed or theoretically developed. In practice, clinicians relied on informal knowledge acquired during training, as described by a senior consultant: *“While we don’t have a formally written guideline, doctors follow the PUO protocol learned during their medical training.”* The perceived need for nationally harmonized PUO protocols was reinforced by the participants’ repeated emphasis that the lack of uniform guidelines led to wide variance in clinical practice across departments and facilities.

#### Organizational Structure and Service Delivery

Variation was observed in how hospitals organized clinical services for PUO management (Table 4). Less than half of the hospitals had a dedicated fever clinic or an infectious disease department. Most hospitals indicated that critical care services, including access to intensive care units, oxygen supply, and backup power, were available during periods of high PUO caseload. Approximately two-thirds of hospitals reported using triage and screening procedures to identify PUO or prolonged febrile illness.

These quantitative findings were supported by qualitative accounts highlighting heterogeneity in service organization. None of the surveyed hospitals used specialized institutional units to handle PUO cases. Instead, depending on their age, patients were treated in pediatric or general medicine departments. A senior pediatrician explained that *“we don’t have a separate ‘Infectious Diseases Unit’ as such. PUO is handled by General Pediatrics.”* Similarly, a hospital administrator from another site noted, *“We manage PUO cases in the general medicine department. We don’t have any separate infectious disease or fever clinic.”*

Although some hospitals had established fever clinics during the COVID-19 pandemic, these were discontinued once the emergency subsided. As one administrator described, *“During COVID, we had a fever clinic, but now fever cases are managed like all other OPD (Outpatients Department) patients.”* Infectious disease expertise existed largely on an informal or consultative basis and was often dependent on specific individuals rather than formally designated focal persons or teams. Multidisciplinary coordination occurred through ad hoc consultations with subspecialties such as oncology, neurology, and gastroenterology, particularly for complex or prolonged cases. However, such coordination was not embedded within a structured PUO care pathway, limiting consistency, efficiency, and continuity of care.

#### Referral Systems

Formal referral systems for PUO management were limited across study sites (Table 4). Only a small proportion of hospitals reported having structured referral mechanisms for advanced diagnostics. Qualitative interviews reinforced these findings, with participants frequently describing reliance on external laboratories for specialized investigations due to in-house diagnostic limitations. Referral processes were often described as informal, ad hoc, and dependent on personal networks rather than standardized institutional pathways. As one infectious disease consultant stated*, “There are no formal MOU (Memorandum of Understanding); referrals depend on the clinician’s decision.”*

For specialized diagnosis or care, patients were routinely referred to higher institutions in Kathmandu or, occasionally, to hospitals in India. Logistical and financial factors frequently impacted these choices. A hospital administrator noted that *“sometimes it is easier and cheaper to send samples to India than to Kathmandu.”* The participants’ reliance on personal professional networks for referrals raised concerns about equity, continuity of care, and system-level coordination.

### Diagnostic and Laboratory Capacity

#### Diagnostic Capacity and Laboratory Readiness

Assessment revealed substantial variability in diagnostic and laboratory capacity for PUO management across the participating hospitals (Table 5). Molecular diagnostic capability, including polymerase chain reaction (PCR) testing, was available in just over half of the hospitals, while a smaller proportion relied exclusively on GeneXpert platforms, which allow rapid detection of specific pathogens but are limited to pre-defined assays and cannot identify a broad range of etiologies. Consequently, reliance solely on GeneXpert restricts the ability to diagnose uncommon or novel causes of PUO. All hospitals reported having an on-site microbiology laboratory. Cold-chain infrastructure was widely available, with most hospitals reporting access to −20°C freezers and a majority having ultra-low temperature (−80°C) freezers for specimen storage. While all laboratories were capable of basic culture and phenotypic testing, including bacterial growth on standard media and routine antimicrobial susceptibility testing, there was considerable variability in the range and complexity of assays performed. Some laboratories offered extended bacterial panels, specialized fungal cultures, and selective media for less common pathogens, whereas others were limited to standard bacterial culture and antimicrobial susceptibility testing. Despite this, dependence on external laboratories for advanced diagnostics was common. More than half of the hospitals referred samples to the National Public Health Laboratory (NPHL), and several others relied on research institutions for specialized testing. These referrals typically included molecular diagnostics such as PCR-based pathogen detection, serological assays, and other specialized microbiological tests that were not routinely available on-site.

Availability of infection prevention resources varied. Approximately half of the hospitals reported having adequate personal protective equipment (PPE) and isolation capacity for PUO cases of suspected infectious origin, while a larger proportion reported a reliable supply chain for diagnostic reagents used in microbiology, serology, and molecular testing for PUO.

Qualitative findings supported and expanded upon these quantitative observations. Participants consistently reported that basic diagnostic investigations, including complete blood counts, inflammatory markers, routine biochemistry, serological testing for common endemic infections, and conventional cultures, were routinely available. On the other hand, access to advanced diagnostics was said to be unreliable and often depended on being referred by someone else. Clinicians commonly described using a stepwise exclusion approach to PUO diagnosis, beginning with common viral and endemic causes and progressing to less frequent autoimmune or malignant etiologies when initial investigations were inconclusive. As one pediatric consultant explained, “*If all these are ruled out, we go up to bone marrow if needed*.”

All facilities have inadequate advanced diagnostic services despite this baseline capacity. Molecular diagnostics, such as multiplex diagnostic panels and PCR-based assays for viral, rickettsial, and fungal diseases, were largely unavailable internally. An infectious disease consultant highlighted this gap, stating, “*We have limited in-house tests. We lack PCR tests for many viral etiologies and rickettsial diseases*.”

As a result, medical professionals often relied on other laboratories, including the National Public Health Laboratory (NPHL), private labs, or Indian facilities. Long hospital stays and delayed diagnoses were common outcomes of this dependence. When diagnoses remained challenging, laboratories reported having sample storage equipment, such as −80°C freezers, in accordance with NPHL guidelines. A laboratory supervisor explained, “*When diagnosis remains elusive, samples are preserved at minus 80 degrees following NPHL protocol*.” None of the hospitals reported the use of standardized PUO diagnostic workflows or predefined test panels. Instead, test selection was primarily guided by individual clinician judgment, as confirmed by a laboratory supervisor who stated, *“There are no specific test bundles (panels) for PUO.”*

#### Surveillance, Reporting, and Digital Systems

Quantitative findings indicate variable use of digital systems for PUO surveillance and reporting across study sites (Table 5). A hospital-wide electronic health record (EHR) or hospital management information system (HMIS) was in use in approximately two-thirds of hospitals. A similar proportion reported routinely communicating PUO-related updates or case alerts to hospital staff. Only a few hospitals reported PUO cases through mechanisms beyond the mandated provincial or national channels (DHIS2), such as reporting trends in increasing fever cases, contributing to variability in surveillance practices across hospitals.

Functionality of existing EHR systems varied. Only a minority of hospitals reported complete documentation of PUO clinical pathways, laboratory findings, and referrals within electronic systems, while partial or no documentation was common. Traceability of PUO cases for follow-up or audit was reported in fewer than half of hospitals, with follow-up activities generally conducted internally within the hospital rather than by local health departments. Routine analysis of clinical and laboratory data to identify PUO or infectious disease patterns was uncommon, with most hospitals reporting that such analyses were conducted only occasionally or not at all. When performed, analyses focused primarily on hospital-level trends and clusters among admitted patients, rather than broader regional disease surveillance. Infrastructure limitations were widely reported as a barrier to adopting or expanding digital systems for PUO management, stemming from factors such as high costs, limited availability of equipment, and insufficient skilled personnel to implement and maintain these systems.

Qualitative findings were consistent with these quantitative patterns. Participants reported that existing digital systems were primarily designed for routine clinical care and not optimized for tracking undiagnosed febrile illnesses. PUO cases were rarely recorded as a distinct entity, and no dedicated registers or dashboards were described. A hospital administrator explained, “We *only report notifiable diseases. There is no separate reporting system for PUO*.” Notifiable diseases include those reported through the national Early Warning Alert and Response System (EWARS), which covers 18 selected priority diseases, 8 syndromes, and public health events.

Most hospitals operated hybrid record-keeping systems, in which hospital-wide electronic health records (EHR) or hospital management information systems (HMIS) were in place, but routine clinical documentation at the provider level remained largely paper-based. Several hospitals reported that existing electronic systems were primarily used for registration, billing, or aggregate reporting rather than for comprehensive clinical documentation. Hospital leadership at one site stated, “We *currently do not have an EMR system, but we are planning to implement it*,” referring to the absence of a fully integrated, clinician-facing electronic medical record. Despite being perceived as potentially beneficial, telemedicine, including infectious disease expert consultation, remained underdeveloped, largely due to limited availability of trained human resources. A senior clinician noted that “*for telemedicine in infectious diseases, we haven’t set it up; there’s no dedicated human resource*,” while another consultant added that “*if we could have tele-consultations once or twice a month with PUO experts, management would be easier*.”

### Human Resources and Infrastructure

#### Human Resources and Technical Expertise

Hospitals differed in the availability of human resources and training pertinent to PUO management (Table 6). Staffing shortages affecting PUO management were reported in eight of eleven hospitals (72.7%). Infectious disease specialists were available in less than half of hospitals (45.5%), whereas internal medicine physicians and resident doctors were more widely represented. All hospitals had medical officers and laboratory technicians, and most hospitals employed ICU-trained nurses, microbiologists or pathologists, and biomedical engineers. Staff trained in molecular diagnostic techniques were present in just over half of the hospitals (54.5%). Although all hospitals conducted regular continuing medical education (CME) or clinical update sessions, only three (27.3%) reported sessions specifically focused on undiagnosed febrile illnesses or PUO in the past six months.

Qualitative findings reinforced these quantitative patterns. Participants highlighted that while core clinical and laboratory personnel were generally available, gaps in specialized expertise, particularly in infectious diseases and molecular diagnostics, constrained PUO investigation. A senior pediatrician emphasized this gap, noting, *“If we had someone with a pediatric infectious diseases fellowship, that would be excellent. We don’t have such a person.”* This perspective reflects challenges specific to pediatric referral settings, where PUO evaluation requires age-specific diagnostic expertise and tailored clinical management. Laboratory technicians without specialized supervision often worked in microbiology services. This was seen as a major drawback, especially when it came to complicated diagnostic interpretation. A laboratory supervisor from one hospital stated, *“Microbiology is run by lab technicians. We need at least one microbiologist.”*

Additionally, participants reported that a lack of skilled staff on weekends, public holidays, and at night caused diagnostic delays for admitted PUO patients. There were few possibilities for PUO-specific training; cases were rarely covered by specialized programs and infrequently discussed in general Continuing Medical Education (CME) sessions. Continuity and institutional memory were further compromised by high employee turnover and the transfer of qualified experts outside. As one hospital administrator explained, *“Many nurses and doctors go abroad, which causes a shortage of skilled manpower and a lack of stability.”*

#### Physical Infrastructure and Bed Capacity

Hospital infrastructure and bed capacity also varied across study sites (Table 6). Approved bed capacity ranged from fewer than 100 to more than 500 beds, with most hospitals having 100–500 beds. General medical and surgical bed availability reflected similar variation. Among the nine hospitals reporting inpatient department (IPD) beds excluding maternity, four had 101–299 beds, three had fewer than 100 beds, and two had more than 300 beds. Adult intensive care unit (ICU) and high-dependency unit (HDU) beds were available in most hospitals, typically ranging from 5 to 14 beds, while pediatric ICU beds were present in six of ten hospitals with a median of four to ten beds.

Infrastructure limitations, particularly related to critical care capacity, were reported across all study sites. Participants described recurrent shortages of ICU beds, including PICU and NICU beds, despite incremental expansions. A senior clinician noted that *“adding two or three ICU beds is not enough; patients arrive from all over the country,”* highlighting the mismatch between service demand and available capacity.

Seasonal increases in febrile illness further strained the capacity of the general ward. Although several hospitals had ambitious plans to expand their infrastructure, including new buildings and specialized units, financial and administrative limitations frequently caused implementation to be delayed. Hospital leadership at one site described these uncertainties, stating, *“We had planned a 1,000-bed hospital, but the current status is uncertain.”* Limited laboratory space was also identified as a barrier to introducing advanced molecular and sequencing-based diagnostics.

### Cross-Cutting Barriers and Future Priorities

#### Financial and Economic Barriers

Financial and economic constraints were widely reported as barriers to effective PUO diagnosis and management across study hospitals (Table 7). High diagnostic costs were identified as a barrier in nine hospitals (81.8%), while infrastructure limitations affecting the adoption of advanced or digital diagnostics were reported by ten hospitals (90.9%). Eight hospitals (72.7%) indicated that lack of staff training, often linked to limited financial resources, further constrained diagnostic capacity.

Similarly, economic constraints were identified as a major determinant of diagnostic decision-making for PUO in qualitative findings. While basic investigations were generally affordable, advanced diagnostics, particularly those requiring referral to external laboratories, imposed substantial financial burdens on patients. A senior clinician explained that “many *patients cannot afford external tests when referred outside the hospital.”*

Referrals to private laboratories raised ethical issues among participants, especially when it came to individuals from low-income backgrounds. Financial barriers often caused delays in diagnosis, which led to longer hospital stays and more out-of-pocket expenditure. As one infectious disease consultant described, *“Testing prolongs hospital stays, increases expenses, and adds financial stress on families.”*

#### Clinical Management and Empirical Treatment Practices

Certain aspects of clinical management practices and challenges, such as empirical treatment strategies, prior antibiotic use, and concerns regarding antimicrobial resistance, were not captured by structured questions in our quantitative survey, even though it collected structured data on hospital infrastructure, staffing, laboratory capacity, and service organization. These conclusions are presented separately as qualitative findings since they came from open-ended qualitative interviews with doctors.

Clinicians often used empirical treatment methods when there was ongoing diagnostic uncertainty. These included doxycycline or azithromycin for suspected rickettsial infections, broad-spectrum antibiotics, and empirical anti-tuberculosis treatment when other causes were ruled out. An infectious disease consultant noted that “*if all investigations are negative, we often suspect tuberculosis and start empirical anti-TB treatment*.”

Prior use of over-the-counter antibiotics before hospital presentation was widely reported and recognized as a major complicating factor for diagnosis. A senior consultant explained that *“by the time patients reach us, most have already taken antibiotics.”* Concerns regarding antimicrobial resistance were frequently raised, particularly in relation to repeated empirical antibiotic use in undiagnosed PUO cases.

#### Future Directions and System Strengthening Priorities

There was broad agreement among all responder categories about the necessity of improved diagnostics, especially metagenomic next-generation sequencing (mNGS), as a potentially revolutionary tool for PUO management. Participants emphasized that such capacity would be most feasible if established at centralized national or provincial reference laboratories, rather than at individual hospitals, allowing specimens to be referred for advanced analysis. At the same time, stakeholders acknowledged technical and cost-related challenges associated with sequencing-based approaches. A senior clinician emphasized that “if *we can introduce genome sequencing and generate Nepal’s own PUO data, that would help build a stronger program*.”

Additionally, participants emphasized the significance of developing telemedicine platforms, creating Nepal-specific evidence to guide policy and practice, and setting up specialized infectious disease or PUO clinics. Despite persistent resource constraints, senior leadership framed PUO as an opportunity for system innovation, with one vice-chancellor stating, “We *see these challenges as opportunities to develop new systems and approaches*.”

## Discussion

This mixed-methods study provides a comprehensive assessment of the institutional readiness and diagnostic challenges for managing PUO across tertiary hospitals in Nepal. The findings reveal substantial gaps across governance, organizational structures, diagnostic capacity, human resources, referral mechanisms, and digital systems. Together, these weaknesses contribute to fragmented diagnostic pathways, reliance on empirical management, and limited surveillance of prolonged febrile illness.

The primary finding was the critical lack of national or hospital-level PUO guidelines, which led to clinician-dependent diagnosis processes shaped by individual experience, contextual epidemiology, and available investigations. Similar observations have been reported in other settings, where PUO lacks universally accepted diagnostic algorithms and clinicians rely heavily on judgment and sequential exclusion strategies [5, 30, 31]. However, the lack of formal guidance in Nepal appears to exacerbate practice variability, particularly among junior clinicians increasing the risk of delayed diagnosis and inefficient use of resources. In high-income settings, structured PUO frameworks emphasize early multidisciplinary evaluation and systematic exclusion of common causes [1]. While such frameworks are not universally standardized even in these contexts, their absence in Nepal underscores the need for context-specific guidance that integrates local epidemiology, endemic infections, and available diagnostics [32].

Organizationally, PUO cases were predominantly managed within general medicine or pediatric units, with limited availability of dedicated infectious disease services or fever clinics. This reflects broader structural constraints in LMICs’ health systems, where specialized services are often underdeveloped due to workforce and resource limitations [32]. Evidence from outbreak settings suggests that dedicated fever clinics can improve triage, coordination, and early diagnosis [33]. In the present study, reliance on informal coordination mechanisms was common but vulnerable to staff turnover and workload pressures, potentially undermining continuity of care.

Consistent with regional and global literature, limited access to advanced diagnostics emerged as a major barrier to PUO management [3, 34, 35]. While basic laboratory services were universally available, access to molecular diagnostics, expanded serological panels, and specialized testing including sequencing approaches was inconsistent and often dependent on external laboratories. These gaps mirror findings from neighboring South Asian countries, where a substantial proportion of PUO cases remain undiagnosed due to diagnostic constraints [36]. Human resource limitations further compounded these challenges. Shortages of infectious disease specialists, microbiologists, and personnel trained in molecular techniques were common, aligning with global evidence of inequitable distribution of skilled health workers in low-resource settings [37]. The absence of structured, PUO-focused continuing professional development limited opportunities to standardize practice and update diagnostic strategies. Previous studies have shown that specialist involvement and structured antimicrobial stewardship programs can improve care quality and diagnostic precision in complex febrile illnesses [1].

Empirical antibiotic use, delayed presentation, and prior treatment before referral were frequently described barriers to definitive diagnosis. While such empirical approaches can be lifesaving in acute settings, they may also increase the risk of adverse drug reactions, contribute to antimicrobial resistance, and complicate the interpretation of subsequent diagnostic results [38, 39]. Pre-hospital antibiotic exposure is known to obscure microbiological findings and complicate PUO workups [40, 41]. In Nepal, this challenge is exacerbated by the widespread availability of antibiotics without prescription and their frequent use through community pharmacies and informal healthcare providers, resulting in many patients receiving partial or inappropriate antimicrobial therapy prior to hospital presentation [42, 43]. In tuberculosis-endemic regions such as South Asia, extrapulmonary tuberculosis remains a leading but diagnostically challenging cause of PUO, often requiring advanced investigations that are not routinely accessible [34, 44]. These factors contribute to the persistently high proportion of undiagnosed PUO cases reported in systematic reviews [4]. Poor coordination between clinicians and laboratories and the absence of formal multidisciplinary case review mechanisms further impeded diagnostic resolution. Evidence suggests that structured clinico-microbiological and clinico-radiological discussions can improve diagnostic yield and reduce unnecessary investigations [45]. Their limited use in the present study reflects missed opportunities for collaborative problem-solving in PUO care, which may perpetuate reliance on empirical management strategies described earlier, particularly in the absence of timely or definitive diagnostic results.

Digital health infrastructure for PUO surveillance was underdeveloped across study sites. Although many hospitals used EHR or HMIS platforms, these systems were rarely optimized for tracking undiagnosed febrile illnesses. PUO cases were seldom recorded as a distinct category, limiting follow-up, audit, and public health reporting. Similar challenges have been reported in other LMIC settings, where digital tools are underutilized for complex disease surveillance [46, 47]. Strengthening digital integration, including interoperable EHRs, decision-support tools, and teleconsultation platforms could improve continuity of care and facilitate referral and follow-up. PUO represents an important sentinel condition for broader surveillance of emerging and re-emerging infections, antimicrobial resistance, and epidemic preparedness.

The strong consensus among participants on the need for national PUO guidelines, advanced diagnostics, dedicated infectious disease services, and structured training aligns with global health system strengthening priorities [37]. Context-specific PUO protocols, integration of molecular diagnostics, and institutionalized multidisciplinary reviews could improve diagnostic yield and reduce unnecessary empirical treatment [4, 48]. In Nepal, only limited capacity development in pathogen sequencing has occurred, largely concentrated in academic and reference institutions such as Center for Infectious Disease Research and Surveillance (CIDRAS) at Dhulikhel Hospital, Kathmandu University Hospital. Sequencing platforms are primarily used for specific research purposes, although these services are not linked to routine clinical PUO diagnostics. Aligning these efforts with digital reporting systems and workforce development could transform PUO management from a reactive clinical challenge into a proactive, data-driven public health strategy. Such reforms are consistent with national health sector priorities for evidence-based care and system resilience [49].

This study provides comprehensive assessments of institutional readiness for PUO management in Nepal using a mixed-methods approach. However, several limitations should be acknowledged. The purposive sampling of 11 tertiary care hospitals, representing only a subset of tertiary-level institutions nationwide, limits generalizability, particularly to secondary- and primary-level facilities. In addition, hospitals from six of Nepal’s seven provinces were included, providing broad national coverage, although province-specific health system contexts from one province were not captured. The cross-sectional design precludes assessment of temporal changes. Qualitative findings may be subject to reporting bias, and patient-level outcomes were not evaluated, limiting direct linkage between institutional readiness and clinical outcomes. Prospective implementation research is needed to evaluate the impact of context-specific guidelines and advanced diagnostics on PUO outcomes. Integration of genomic surveillance with clinical decision-making, supported by workforce development and digital infrastructure, could transform PUO management from reactive to proactive, data-driven care.

## Conclusions

The study demonstrates substantial gaps in governance, diagnostic capacity, infrastructure, human resources, referral mechanisms, and digital health capabilities that limit the management of PUO in tertiary hospitals in Nepal. In the absence of standardized national or institutional guidelines and formal referral pathways, PUO care remains highly clinician-dependent, with frequent reliance on external laboratories for advanced diagnostics. Financial, workforce, and infrastructure limitations further impede timely and equitable diagnosis and management. Despite these challenges, hospitals exhibited important strengths, including the availability of basic diagnostic services, engagement in continuing clinical education, and access to critical care for patients with severe or prolonged febrile illness. Addressing identified gaps through the development of context-specific national guidelines, targeted workforce training, strengthened laboratory and digital infrastructure, and coordinated policy interventions could substantially improve institutional readiness, standardize PUO management, and enhance patient outcomes in Nepal.

## Figures and Tables

**Table 1.** Domains and Key Indicators Used to Assess Institutional Readiness and Management Capacity.

S.N.	Domain	Indicator
1	Institutional Readiness	Diagnostic infrastructure, human resources capacity, clinical protocol availability, functional referral pathways, and sample storage/transport mechanisms.
2	Diagnostic Practices	Operational PUO definitions, diagnostic pathways followed (e.g., CBC, malaria RDT, blood culture, imaging sequences), empirical treatment patterns (e.g., antibiotics/antitubercular therapy), and documentation practices.
3	Challenges/Barriers	Technical limitations (equipment/test availability), logistical constraints (supply chain, result delays, transport), human resource gaps, financial barriers, and systemic/policy-related issues (absence of national guidelines, poor diagnostic-clinical integration) all play a significant role in healthcare.
4	Stakeholder Perceptions	Feasibility and utility of advanced diagnostics (metagenomic sequencing, nanopore technology), willingness to adopt new platforms, and identified capacity-building needs.

**Table 2.** Themes and Codes for Qualitative Analysis

Theme	Subtheme	Codes
**Governance & Policy**	National & Institutional Guidance	Absence of national PUO guidelines; reliance on international textbooks; hospital SOPs informal or nonexistent; need for standardized protocols.
	Clinical Decision-Making	Individualized decision, clinician-driven management.
**Service Delivery & Organization**	PUO-Specific Units	Lack of dedicated PUO/fever clinics; absence of formal infectious disease units
	Multidisciplinary Coordination	Informal focal persons, ad hoc team consultations, and unstructured care pathways
	Critical Care Support	Inconsistent ICU/PICU availability; seasonal bed pressures
**Diagnostics & Laboratory Capacity**	Basic & Advanced Tests	Basic diagnostics are widely available; limited molecular/advanced testing and a stepwise diagnostic approach
	Test Standardization & External Dependence	Absence of PUO test bundles, reliance on external labs, and variable sample storage capacity
**Human Resources & Expertise**	Clinical Staff	Shortage of pediatric ID specialists; uneven availability of microbiologists/pathologists; staffing gaps
	Training & Knowledge	Limited PUO-specific training or CME; workforce attrition; skill retention challenges
**Infrastructure & Facility Readiness**	Beds & ICU	ICU/PICU shortages; few PUO-specific beds; seasonal bed demand
	Laboratory & Facility Space	Limited lab space for advanced diagnostics; delayed infrastructure expansion
**Referral Systems**	Diagnostic Referral	Limited formal referral systems; reliance on NPHL, research institutes, or cross-border labs
	Coordination & Equity	Informal clinician-dependent pathways; equity and continuity-of-care concerns
**Financial & Economic Issues**	Costs & Access	High cost of advanced/external diagnostics; patient affordability issues; prolonged hospital stays expenses
	Training & Infrastructure	Financial limitations restricting training; infrastructure constraints linked to funding
**Clinical Management &**	Empirical Treatment	Use of antibiotics, anti-TB therapy; prior community antibiotic use complicates diagnosis
**Empirical Practices**	Antimicrobial Resistance	Risk due to repeated empirical treatment; individualized clinical decisions due to lack of standard guidance
**Surveillance & Digital Systems**	Reporting & Tracking	PUO not routinely captured; weak case tracking/follow-up; limited EMR/EHR functionality
	Telemedicine & Digital Health	Underdeveloped tele-ID services; potential for teleconsultation
**Future Directions & System Strengthening**	Diagnostics & Research	Need for advanced diagnostics (e.g., mNGS); Nepal-specific PUO evidence
	Service & Policy Development	Dedicated PUO/ID centers, teleconsultation platforms, policy engagement, and evidence-to-practice translation

**Table 3.** Characteristics of Study Sites and Key Informant Participants

Characteristic	Value
**Study sites (hospitals) (N = 11)**	n (%)
Koshi Province	1 (9.1)
Madhesh Province	1 (9.1)
Gandaki Province	1 (9.1)
Lumbini Province	1 (9.1)
Karnali Province	2 (18.2)
Bagmati Province	5 (45.5)
**Type of hospital (N=11)**	n (%)
Public, academic	6 (54.5)
Public, non-academic	3 (27.3)
Community	1 (9.1)
Private, academic	1 (9.1)
**Key Informant Interviews (KIIs) (N = 33)**	n (%)
Male participants	31 (93.9)
Female participants	2 (6.1)
In-person interviews	18 (54.5)
Online interviews	15 (45.5)
**Mean years of professional experience (N=33)**	Mean years
Hospital directors/administrators (n=11)	11.57
Infectious disease / general medicine doctors (n=11)	8.15
Laboratory technologists/microbiologists (n=11)	11.9

**Table 4.** Health System Readiness for PUO Management

Characteristics	Frequency (n)	Percentage (%)
**Institutional Preparedness and Structural Capacity**		
PUO-specific clinical guidelines or SOPs available	1	9.1
PUO-specific response drills or simulations conducted	1	9.1
SOP for internal coordination among departments for PUO	0	0
Designated PUO focal person or committee	3	27.3
**Organizational Structure and Service Delivery**		
Infectious Disease Department	5	45.5
Fever clinic in the hospital	4	36.4
PUO cases managed within general medicine/pediatrics	11	100.0
Critical care services (e.g., ICU, oxygen, power supply) were ensured for PUO patients during high caseload.	9	81.8
Triage and screening procedures established for identifying PUO or prolonged febrile illness cases	7	63.6
**Referral Systems**		
Formal referral system for advanced diagnostics	3	27.3
External referral: NPHL	6	54.5
External referral: Research Institutes	3	27.3

**Table 5:** Diagnosis and Laboratory Capacity for PUO Management

Indicator	Frequency (n)	Percentage (%)
**Diagnostic capacity and laboratory readiness**		
Molecular testing capacity (PCR available)	6	54.5
Only GeneXpert	2	18.2
On-site microbiology laboratory	11	100.0
- 80°C freezer for sample storage	8	72.7
- 20°C freezer availability	10	90.9
External lab dependence (NPHL)	6	54.5
External lab dependence (other research institutes)	3	27.3
Adequate PPE and isolation capacity for PUO cases of suspected infectious origin	6	54.5
Reliable supply chain for PUO-related diagnostic reagents	8	72.7
**Surveillance, reporting, and digital systems**		
Hospital-wide EHR/HMIS system in use	7	63.6
Formal mechanism (excluding DHIS2) to report PUO cases to public health authorities	2	18.2
PUO updates or case alerts are regularly communicated to hospital staff	7	63.6
EHR captures PUO clinical pathways, lab results, and referrals		27.3
Yes	3	
Partially	5	45.5
No	3	27.3
PUO cases traceable for follow-up/audit	4	36.4
Formal mechanism (excluding DHIS2) to report PUO cases to public health authorities	2	18.2
PUO updates or alerts are regularly communicated to hospital staff	7	63.6
The hospital analyzes clinical/laboratory data to identify PUO or ID patterns		18.2
Yes	2	
Occasionally	7	63.6
No	2	18.2
Infrastructure limitation as a barrier to adopting digital diagnostics for PUO management	10	90.9

Abbreviations: NPHL - National Public Health Laboratory; EHR - Electronic Health Record; HMIS - Health Management Information System; DHIS2 - District Health Information Software 2.

**Table 6.** Human Resources and Infrastructure (N = 11)

Indicator	Frequency (n)	Percentage (%)
Staffing challenges affecting PUO management	8	72.7
Infectious disease specialists are available	5	45.5
Internal Medicine Physicians	9	72.7
Medical officers	11	100
Resident doctors	9	72.7
ICU-trained nurses available	9	72.7
Microbiologists/Pathologists	10	90.9
Lab technicians	11	100
Biomedical Engineers	8	72.7
Staff trained in molecular techniques	6	54.5
Hospitals conducted regular Continuing Medical Education (CME) or clinical update sessions	11	100
Any CME or clinical sessions focused on undiagnosed febrile illness or PUO have been conducted in the last 6 months	3	27.3
**Physical infrastructure and bed capacity**		
Approved bed capacity		
100–300 beds	5	45.5
300–500 beds	3	27.3
>500 beds	3	27.3
General beds (medical + surgical)		
≤100 beds	4	36.4
101–699 beds	5	36.4
≥700 beds	3	27.3
IPD beds (excluding maternity) (n = 9)		
≤100 beds	3	33.3
101–299 beds	4	44.5
≥300 beds	2	22.2
Adult ICU beds		
None	1	9.1
5–14 beds	6	54.5
>15 beds	4	36.4
Adult HDU beds		
None	1	9.1
5–12 beds	6	54.5
≥13 beds	4	36.4
Pediatric ICU beds (n = 10)		
None	2	20
4–10 beds	6	60
>10 beds	2	20

**Table 7.** Financial and Economic Barriers to PUO Diagnosis and Management

Indicator	Frequency	Percent
Cost as a barrier to adopting digital/advanced diagnostics for PUO	9	81.8
Infrastructure limitation as a barrier to PUO diagnostics	10	90.9
Lack of training as a barrier (financially linked)	8	72.7

## Data Availability

The data collection tools used in this study are provided as supplementary materials. De-identified datasets and additional study materials will be made available by the corresponding author upon reasonable request.

## List of abbreviations

AMR: Antimicrobial Resistance
EHR: Electronic Health Record
FUO: Fever of Unknown Origin
KII: Key Informant Interview
PCR: Polymerase Chain Reaction
PUO: Pyrexia of Unknown Origin
SOP: Standard Operating Procedure
